# COVID‐19 and anatomy: Stimulus and initial response

**DOI:** 10.1111/joa.13274

**Published:** 2020-07-06

**Authors:** Cecilia Brassett, Thomas Cosker, D. Ceri Davies, Peter Dockery, Thomas H. Gillingwater, T. Clive Lee, Stefan Milz, Simon H. Parson, Fabio Quondamatteo, Tracey Wilkinson

**Affiliations:** ^1^ Department of Physiology Development and Neuroscience University of Cambridge Cambridge UK; ^2^ Department of Physiology, Anatomy and Genetics University of Oxford Oxford UK; ^3^ Human Anatomy Unit Department of Surgery and Cancer Imperial College London London UK; ^4^ Department of Anatomy School of Medicine National University of Ireland Galway Galway Ireland; ^5^ Edinburgh Medical School: Biomedical Sciences University of Edinburgh Edinburgh UK; ^6^ Department of Anatomy and Regenerative Medicine Royal College of Surgeons, Ireland University of Medicine and Health Sciences Dublin 2 Ireland; ^7^ Anatomische Anstalt Ludwig‐Maximilians‐Universität München Munich Germany; ^8^ Department of Anatomy Suttie Centre School of Medicine Medical Sciences and Nutrition University of Aberdeen Aberdeen UK; ^9^ Anatomy Facility University of Glasgow School of Life Sciences Glasgow UK; ^10^ Centre for Anatomy and Human Identification School of Science and Engineering University of Dundee Dundee UK

**Keywords:** anatomy, body donation, coronavirus, COVID‐19, education

## Abstract

The outbreak of COVID‐19, resulting from widespread transmission of the SARS‐CoV‐2 virus, represents one of the foremost current challenges to societies across the globe, with few areas of life remaining untouched. Here, we detail the immediate impact that COVID‐19 has had on the teaching and practice of anatomy, providing specific examples of the varied responses from several UK, Irish and German universities and medical schools. Alongside significant issues for, and suspension of, body donation programmes, the widespread closure of university campuses has led to challenges in delivering anatomy education via online methods, a particular problem for a practical, experience‐based subject such as anatomy. We discuss the short‐term consequences of COVID‐19 for body donation programmes and anatomical education, and highlight issues and challenges that will need to be addressed in the medium to long term in order to restore anatomy education and practice throughout the world.

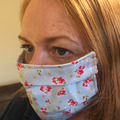

## 1 INTRODUCTION

The emergence of infectious diseases with the potential to spread rapidly among the human population, such as severe acute respiratory syndrome (SARS) and Zika virus, presents a major threat to global public health (Wolfe *et al*., 2007; Ventura *et al*., 2016). However, the recent arrival of a severe respiratory disease, first reported in Wuhan city (Hubei province) China in December 2019 (Wu *et al*., 2020, Zhu *et al*., 2020), has generated an unprecedented global response. COVID‐19 is caused by a novel and highly pathogenic coronavirus (SARS‐CoV‐2) that is easily and rapidly transmitted through respiratory droplets (Wu *et al*., 2020, Zhu *et al*., 2020).

The highly contagious nature of COVID‐19, and the potentially life‐threatening nature of symptoms for an affected individual, has led to widespread, global attempts to control person‐to‐person contact and disease spread within and between populations (Wilder‐Smith *et al*., 2020). One major consequence of the ensuing political decisions has been a closure of most university campuses, and with them medical schools and anatomy units/departments.

In the following review of actions taken after the COVID‐19 outbreak, information was collated from the following 10 universities:

Ireland: National University of Ireland Galway (NUIG), Royal College of Surgeons in Ireland (RCSI).

Scotland: Universities of Aberdeen, Dundee, Edinburgh and Glasgow.

England: University of Cambridge, Imperial College London, University of Oxford.

Germany (Bavaria): Ludwig Maximilian University of Munich (LMU).

Although there is no a priori rationale for claiming these universities are representative of all affected European universities, the similarities of their response to the COVID‐19 pandemic suggest that in this respect at least, they might be. The challenges that have arisen, and/or are likely to arise, over the coming months and years are outlined, and the immediate decisions taken, the rationale for them and their effects are described on an institution‐by‐institution basis. In the final section, possible longer‐term effects and challenges are also discussed.

## 2 CLOSURE OF UNIVERSITY CAMPUSES, MEDICAL SCHOOLS AND ANATOMY UNITS/DEPARTMENTS IN RESPONSE TO COVID‐19

### 2.1 Aberdeen

The Principal of the University announced University‐wide lockdown on 13 March, to be in place by 20 March. This affected teaching sessions for *c*.800 undergraduate students on medical, dental, physician associate and science programmes. Fortunately, the majority of practical teaching was able to be completed prior to lockdown, except for Year 3 Medicine students who would have returned after the Easter vacation. The practical element of two, ongoing blended anatomy postgraduate courses was also suspended. At the same time, several surgical skills courses catering for *c*.100 delegates had to be cancelled. Postgraduate (PhD) student work on cadaveric material also ceased. All anatomy staff commenced working from home by 20 March, and the anatomy building closed to all but essential staff, which comprised two licensed teachers and two technicians. The extensive collection of prosected cadaveric material and freezer content was monitored weekly. As the building is shared by the University and the National Health Service (NHS), it was immediately opened for essential training which was coordinated by staff in the Clinical Skills Centre. To date, no requests have been forthcoming for assistance with mortuary facilities. The Anatomy team donated all basic Personal Protective Equipment (PPE) to the NHS. Currently, the University Senate has agreed a fortnight's delay to the start of the first semester in September.

### 2.2 Cambridge

An announcement was made by the Vice‐Chancellor on 18 March that by the end of 20 March, the University's normal operations would cease. The teaching term for all three years of preclinical medical students had already ended on 13 March, with completion of an anatomy practical exam for over 300 second year medical students on that day. Most students returned home on or soon after 13 March, although students who could not do so, including international students and those with immunosuppressed family members, were supported in University/College accommodation where some remain in residence. Clinical teaching for Years 4 & 5 students ceased after 13 March, and final year (Year 6) clinical examinations were cancelled. Several external courses in surgery and ultrasound in regional anaesthesia were also cancelled, affecting *c*.100 delegates. The department was not asked to provide mortuary facilities. A donation of PPE was made to the local hospital following a university‐wide appeal.

### 2.3 Dundee

It was announced on 15 March by the Interim Principal that all face‐to‐face teaching would be suspended from 16 March, with an immediate move to online teaching. The university then closed down for all but essential activities on 18 March. Three weeks of anatomy teaching remained for most students, who comprised *c*.900 undergraduate and postgraduate students in anatomical sciences, forensic anthropology, medicine, dentistry, oral health sciences, and medical and forensic art. Medical students are taught for a longer period, with classes for Year 3 students continuing well into May. However, there was no more dissection in that period, with only some anatomy revision sessions remaining. MSc students carrying out practical projects on Thiel embalmed bodies either completed data collection swiftly before university closure, or moved to literature projects instead. PhD students using Thiel specimens had to suspend data collection for the foreseeable future. All external activities, including surgical skills courses, research projects, service work with medical device companies, and any other events utilising Thiel bodies were suspended. While most staff members commenced working from home on 19 March, technical staff continued to enter the building for essential maintenance, and both technical and administrative staff were permitted to go into work to prepare bodies for cremation. The local Dundee COVID‐19 response teams requested provision of the following: (a) access to dissecting rooms for body storage if the local mortuaries were stretched beyond capacity; (b) assistance from technical staff to ease anticipated pressure on pathologists and technicians at the local police mortuary, where Anatomy staff had already undergone induction, as well as the mortuary at the local teaching hospital; (c) loan of the Anatomy van, which has the capacity to transport six bodies at a time; and (d) provision of any excess PPE as part of a Dundee‐wide initiative.

### 2.4 Edinburgh

All on‐campus teaching at the University of Edinburgh was formally suspended following an announcement from the Principal on 13 March with a move to online teaching and examinations. From 16 March, all face‐to‐face anatomy teaching ceased, affecting *c*.1,000 medical students from Years 1, 2, 4 & 5, as well as *c*.100 Biomedical Science students and 5 postgraduate Master's students. The online Anatomical Sciences postgraduate programme remained unaffected, but with some students requiring extension of deadlines due to changing personal and professional circumstances. All anatomy examinations for Year 1 & 2 Medicine students were cancelled, as were practical spotter examinations for Biomedical Science undergraduates and MSc Human Anatomy postgraduates. In addition, several postgraduate and continuing professional development (CPD) courses had to be cancelled, both at the University and at the Royal College of Surgeons of Edinburgh. Final year Medicine students were permitted to graduate early in April 2020, to allow them to join the NHS workforce dealing with the COVID‐19 pandemic. All Anatomy staff, including academic, technical, museum and research staff, commenced working from home following a meeting on 16 March, except for two senior Anatomy Technicians and the Professor of Anatomy, who retained access to care for donor remains. A request was received from the University of Edinburgh and NHS colleagues in Pathology for technical/mortuary assistance with COVID‐19 research post‐mortems, albeit taking place outside of Anatomy facilities.

### 2.5 Glasgow

The University of Glasgow announced on 14 March that all face‐to‐face teaching would be suspended from 16 March, and that arrangements had to be put in place to deliver online teaching. In advance of this, considerations had already been made regarding cancellations of continuing professional development (CPD) courses. This coincided with the announcement on 13 March that the educational activities of the Royal College of Physicians and Surgeons of Glasgow (RCPSG) would be suspended until the summer. These included courses hosted in the Clinical Anatomy Skill Centre, a joint initiative between the College of Medical Veterinary and Life Sciences of the University of Glasgow and the RCPSG. Only a week of teaching remained for the Life Sciences courses, which comprised *c*.380 students in Year 2 and *c*.80 students in Years 3 & 4. A longer period of teaching was outstanding for the following groups: over 600 medical students in Years 1 & 2; a smaller number in the pre‐medical Glasgow Access Programme; *c*.160 dental students in Years 1 & 2 who receive anatomy teaching in our facility; and *c*.120 nursing students in Years 1 & 2. However, the bulk of gross anatomy teaching and dissection activities had already been completed for these students. In addition, an international Undergraduate group of 12 students taking a Functional Anatomy course had two weeks of teaching remaining, and a postgraduate student cohort, comprising 14 Master's students and 2 undertaking a postgraduate certificate that incorporates Anatomy teaching, still required a substantial amount of teaching. These two groups were most affected by dissection room closure. From the week beginning 23 March, all staff commenced working from home, as the building in which the Anatomy Facility is housed was closed and only accessible for essential maintenance and key workers in case of emergency.

### 2.6 Imperial College London

Imperial College London closed for face‐to‐face formal teaching on 20 March and all staff, apart from key workers, were instructed to work from home. This coincided with the last day of the Spring Term for the Faculty of Medicine and anatomy teaching for preclinical medical undergraduates had already finished, as timetabled. On 17 March, a formative online anatomy spotter examination had already been sat by *c*.360 first year medical students. On 18 and 20 March, 280 medical students in Year 6 sat their final examinations online. A Year 2 Objective Structured Practical Examination for *c*.360 students planned for 19–20 March was cancelled, with all students permitted to progress to Year 3 without sitting the examination. There is no timetabled anatomy teaching for Years 1 & 2 in the Summer Term, and the Year 1 summative anatomy spotter examination will take place online. The small amount of anatomy normally delivered to students in their clinical years is being delivered online for the remainder of the academic year. Imperial College delivers the Core Surgical Anatomy course to *c*.90 first year core surgical trainees (CT1) and Surgical Skills Courses for Specialist Surgical Trainees in the London Postgraduate School of Surgery. The practical elements of these courses have been cancelled until further notice and as much teaching as possible will be delivered online, while bearing in mind the practical limitations of online teaching of surgical skills and the likely reassigning of trainees to non‐surgical front‐line duties. All other postgraduate anatomy courses have also been cancelled. Anatomy facilities were offered to the NHS but were deemed unsuitable for its current needs. The Human Anatomy Unit donated PPE for use by the NHS.

### 2.7 Münich (LMU)

The State of Bavaria closed all state‐run university buildings to students and the general public on 17 March. Employees who had returned within 14 days prior to that date from a region of the world recognised as a high‐risk area by the Robert‐Koch‐Institute Berlin were immediately home quarantined for 14 days. All employees were asked to work from home if possible. On 20 March, the Bavarian government issued a general public contact restriction for all citizens, resulting in further reduction of employee personal contacts within the university sector. From 24 March, all pregnant LMU employees were sent home for the duration of the crisis. Clinical departments prepared for treatment of COVID‐19 patients and other emergency cases. Staff members on the preclinical medical faculty continued working and were tasked with preparing for a summer term of online teaching. All university employees, especially medically trained personnel, were registered for emergency service in the public healthcare sector, potentially depleting the number of anatomy staff able to continue to undertake anatomy education activities.

### 2.8 NUI Galway

On 12 March, the Irish Government announced a range of stringent measures to help combat the spread of COVID‐19, which included the closure of all schools and colleges in the country. Consistent with public health protocols and priorities, campuses were only accessible by those doing work related to, or supporting the public health service in COVID‐19‐specific work. Only key technical staff were permitted access to maintain facilities. There were only 3 weeks left in the NUI Galway term, ending on 4 April in 2020. The lockdown mainly affected medical students taking Gastrointestinal System and Renal System modules, normally delivered as an integrated systems‐based module with lectures from Anatomy, Physiology, Biochemistry and clinical disciplines. Only 60% of the Gastrointestinal System practical sessions were able to be completed, and the Renal system practical did not run. There were similar cancellations of practical classes for undergraduate science students taking Gastrointestinal System and Head and Neck modules. Teaching for biomedical device companies and the MSc/PG Dip in multidisciplinary Radiology was also suspended.

### 2.9 Oxford

In accordance with the UK Government directive, face‐to‐face teaching was suspended on 13 March, which primarily affected students in Years 1‐3 of the undergraduate medicine course. Year 1 did not receive their usual practical classes in the final term, which normally focuses on the lower gastrointestinal tract and genitourinary system. Year 3 students were scheduled to undertake the intensive Principles of Clinical Anatomy course in June, during which they would have daily sessions in the dissecting room revising all aspects of anatomy before proceeding to clinical school. While this course remains under review dependent on further Government directives, extensive preparations were being made to deliver the teaching online if necessary. Graduate Entry Medicine and Biomedical Science students were also significantly affected. Final year medical students graduated early so that they could start helping on the NHS front‐line. The body donation programme was suspended and locked down, with a small number of technical staff permitted to access the building regularly for monitoring of the current donors. The Nuffield Department of Surgery training course was cancelled, during which experienced surgeons would come for an intensive week to undertake cadaveric procedures in their specialty, an invaluable resource for junior surgical trainees.

### 2.10 Royal College of Surgeons in Ireland (RCSI)

An Irish Government directive required the closure of all schools and universities from 12 March. As a result, all campus‐based anatomy teaching ceased for 375 Year 1 medical and physiotherapy students and 15 postgraduate Physician Associate students, who had 4 weeks of teaching term still to run. In addition, a Policy Statement from the Surgical Royal Colleges on 16 March postponed MRCS Examinations and training courses, affecting *c*.200 trainees.

## 3 BODY DONATION PROGRAMMES

### 3.1 Aberdeen

The University of Aberdeen suspended its body donation programme from the evening of 13 March. This decision was taken in conjunction with other anatomy units in Scotland and Her Majesty's Inspector of Anatomy for Scotland (HMIAS). The rationale for this was two‐fold: an inadequate knowledge of the potential risks from COVID‐19 donors to staff, and the increased risk from a continued need to re‐enter the anatomy facility on the Aberdeen Royal Infirmary campus. Planned cremations and burials for March and April were also put on hold. Subsequently, the Scottish Government relaxed the requirement to dispose of donated cadavers within 3 years, extending this by 6 months in the first instance. The annual memorial service for families of donors, which was scheduled for late March, had already been cancelled ahead of the University‐wide closure, as many attendees would be from the vulnerable population. Notification of two bodies was received on 13 March, and these were accepted into the facility on 16 March. While both were elderly and stated to be COVID‐19 negative, they had not been tested for confirmation. The Bequeathal Secretary continued to accept completed bequeathal forms and deal with enquiries while working from home. Import of frozen anatomical material from Science Care USA was suspended, with an offer to hold material for 6 months in the first instance.

### 3.2 Cambridge

Acceptance of body donations ceased on 18 March, but a donor who had already been processed was accepted on 19 March. Teaching of first year medical students, with hands‐on cadaveric dissection, had already been completed in the first two terms (from October 2019 to mid‐March 2020) of the academic year. Therefore, the decision was made to manage the current donors by performing retention of parts with the appropriate consent for future teaching and research, completing cremation paperwork, purchasing coffins and booking cremation slots as soon as possible. This would enable respectful disposal of donors, as well as fulfilling their wishes for the use of their bodies for teaching and research. As these donors had already been dissected by students, their remaining in the dissection room (DR) for a prolonged lockdown would constitute a health and safety hazard. In addition, existing holdings of anatomical prosections needed to be secured, as many were stored in cabinets and required weekly spraying. The decision was made to submerge these prosections in preserving fluid within sealed containers to obviate the need for staff to make regular journeys to the facility. To achieve the above, staff members with access to the DR during the week commencing 23 March were the Bequeathal Secretary and Clinical Anatomist, to complete and check cremation documentation for 42 donors, and subsequently the Senior Technician and Clinical Anatomist on selected days for retention of tissue, encoffining of donors and supervising transport to the crematorium. Cremations were completed by the end of April, at least six weeks earlier than in previous years. The Committal Service for students and staff was delivered online via our secure Virtual Learning Environment (VLE) platform. Students provided a donor tribute from each table group, with two representatives giving general tributes on behalf of the whole cohort. Tributes and biographical material from donors' relatives were also included.

### 3.3 Dundee

From 13 March, increased mortuary health and safety measures were instituted in Dundee, including non‐acceptance of suspected or confirmed COVID‐19 cases, deep cleaning after embalming and full PPE to be worn at all times when accepting bodies for anatomical examination. Due to the submersion of bodies in a Thiel tank for six months, the risk from COVID‐19 was considered to be relatively low, except at the time of embalming. However, on 16 March, HMIAS, after consultation with all the anatomy centres in Scotland and to ensure a consistent approach across the country, suspended all acceptance of body donations until further notice. The Inspector also proposed extending the law compelling cremation or burial of donors within 3 years of their date of death. As this required passing through the Scottish Parliament, the provision was heard and approved as part of the Coronavirus (Scotland) Bill on 1 April. Information on COVID‐19 and the risks associated with the deceased was sparse, except for the Royal College of Pathologists’ guidance for mortuary staff dealing with COVID‐19 bodies (Royal College of Pathologists, 2020; Royal College of Surgeons, 2020). Subsequently, the International Committee of the Red Cross published general guidance on the management of the deceased with COVID‐19 (Finegan *et al*., 2020). In view of the vulnerability of the attendees at our thanksgiving memorial event, due to be held in May, the decision was made to postpone this until the autumn, which would also allow students to attend. The response from both potential donors and relatives of current donors has been one of complete understanding in all respects. Some phoned to ask whether a COVID‐19 patient would be accepted, others to enquire whether the donor programme had been suspended and whether bequeathal forms were still available, and others got in touch with regards to collection of their loved one's ashes. In order to free up the dissecting room and other secure spaces which may be required for body storage due to the current crisis, donor cremations continued.

### 3.4 Edinburgh

Following local discussions with other Scottish Anatomy Departments, the Licensed Teachers took the collective decision to suspend the Anatomy@Edinburgh body donation programme from the evening of the 12 March, with immediate effect. This decision was communicated to HMIAS and senior colleagues at the University of Edinburgh, and was swiftly enacted due to the significant amount of fresh frozen material handled by the facility that does not undergo fixation. Formal notices of the suspension were placed on the Anatomy@Edinburgh website, social media channels, and as an answerphone message on the bequest telephone line. At this stage, to ensure that all donor material coming close to the 3‐year retention rule enforced by the Human Tissue [Scotland] Act 2006 would not be held beyond this period, cremation was prioritised for the longest held donors. At the same time, the decision was taken to postpone the annual Anatomy Memorial Service, due to take place in late April, until a later date. There was sufficient embalmed donor material to meet teaching requirements for the 2020‐21 academic year, although the impact on fresh frozen material, largely used for postgraduate and professional courses, remained unclear.

### 3.5 Glasgow

On the evening of the 12 March, as the crisis began to escalate in the UK, the decision was taken to suspend the acceptance of donors until further notice from the following day. A notice was placed online and on the Bequest Coordinator's telephone answering machine as well as in an out‐of‐office reply to emails. The suspension was based on the following considerations: (a) the unknown COVID‐19 status of each donor, as every donor in a pandemic could be a potential carrier of the infection and this could have presented a high risk to the safety of staff handling the donors and subsequent students; (b) the regular practice to exercise extreme caution to protect against potential infectious diseases; (c) information about the infectivity and virulence of the virus in the deceased was sparse; (d) formaldehyde embalming kills most pathogens and is likely to include SARS‐CoV‐2, but this has not yet been verified; (e) *c*.55% of the donor cohort is normally fresh frozen for use in clinical skill courses, so the potential biocidal benefit of embalming would not apply; and (f) in the present crisis, the potential difficulties faced in communicating with doctors who sign the Medical Certificate of the Cause of Death, in order to inform decisions regarding donor acceptance. On 16 March, official advice was issued from the Scottish Government to suspend all donor‐related activities, including cremations, which was immediately actioned in Glasgow. No information was immediately available concerning the response of donors and relatives regarding suspension of the donor programme. The Donor Commemoration Service in Glasgow, which normally takes place in the autumn, may still go ahead subject to circumstances.

### 3.6 Imperial College London

Imperial College London is part of a consortium, together with Anglia Ruskin University, Brighton and Sussex Medical School, King's College London, Queen Mary University of London, St George's University of London and University College London that runs a common donation programme from the London Anatomy Office (LAO). In view of the fact that the SARS‐CoV‐2 virus that causes COVID‐19 is classified as a Hazard Group 3 pathogen that may survive for days in cold damp conditions (Kampf *et al*., 2020), a decision was taken by the consortium on 12 March not to accept confirmed or suspected COVID‐19 cases for Anatomical Examination. However, that decision was superseded by the closure of the LAO on 18 March on advice from its undertakers who no longer had capacity to transport, store or cremate our donors. The LAO staff continued to respond to telephone calls and emails from donors’ families and potential donors to explain the current situation and postponement of the annual Commemoration Service due to take place on 15 May. The Faculty of Medicine at Imperial College London (ICL) was placed in lockdown on 20 March, which was the last day of the Spring Term. ICL should have sufficient donors for teaching in the academic year 2020‐21, but was unable to release donors for funerals. Although the Human Anatomy Unit was in lockdown, several members of staff, as designated key workers, were permitted access to the facilities to ensure that donor material was maintained in accordance with the provisions of the UK Human Tissue Act (2004).

### 3.7 Münich (LMU)

The body donation programme in Münich was not suspended. However, SARS‐CoV‐2‐infected potential donors were not accepted and incoming donors were subject to a virus test. All cadavers designated for use in anatomical teaching were fixed with a formalin and alcohol solution, and then stored for several weeks in embalming solution prior to dissection. Cadavers for clinical courses in surgical procedures were usually embalmed with an alcohol glycerine solution. Fresh frozen human material is currently only used for research purposes and not for teaching.

### 3.8 Nui Galway

The intake of donations had already been suspended in November 2019 until January 2021 due to storage capacity issues. Sufficient pre‐COVID‐19 donations exist for the next 2 years, based upon current usage. The annual Memorial Service planned for 26 March had already been cancelled on 6 March due to concerns over the potential risk to vulnerable elderly attendees. Monitoring and spraying of prosected specimens contained in cabinets in the department were continued by key workers on the anatomy staff team. Planned burial of donors was suspended until further notice.

### 3.9 Oxford

Acceptance of all new donations was suspended from 13 March with the escalation of the COVID‐19 crisis across the UK. This decision was taken by the Director of Anatomy with the Head of Department. A number of donor families, both current and prospective, were informed prior to the suspension and all understood the reasons for it. The message on the bequeathal answerphone was changed to provide an explanation for the suspension of donations, with information on the procedure to be followed in the event of a donor's death. An emergency contingency planning meeting was held to ensure that all technical staff were conversant with the new procedures during the period of lockdown and a rota was established to ensure that current donors would receive regular and dignified care. An emergency contact system was in place at all times, ultimately leading to the Director of Anatomy, in order that any environmental changes in the dissection room could be managed expeditiously.

### 3.10 RCSI

It was planned to suspend acceptance of body donations due to COVID‐19, but the decision had already been taken two days ahead of the enforced closure, as full capacity for body storage had already been reached. The decision was taken in consultation with College management. Prior to the suspension, information was provided for both current and prospective donor families, who understood the immediate reason and the COVID‐19 situation. A Memorial Service for donors is held every second year and as the next event will be held in 2021, it is unlikely to be affected.

## 4 MOVING TO ONLINE DELIVERY OF ANATOMY EDUCATION

### 4.1 Aberdeen

The immediate priorities were the summer spotter examinations for science and medical students, which had originally been scheduled for March and April. The Medical School had clear plans at all levels at an early stage, but a centralised message from the University did not fit well with healthcare programmes, which have a different tempo and timescale to other university courses. Therefore, considerable work had to be spent on designing online alternatives for the summer examinations. Immediately prior to lockdown, some science course spotters had already been conducted in an electronic format in lecture theatres as usual. A final decision was made to convert all year 1‐3 medical summative spotters into formative assessments. While these were open for 2 weeks to allow for circumstances such as illness, having to care for relatives and differing time zones, each student would be required to select a shorter time frame within this period to take the paper. These results were principally used to identify students who needed additional support. It was accepted that more time was needed to enable students to reach the necessary level of knowledge, and that conventional end of year examinations covering a wider range of material may still be required, with extensive validation from internal quality audit. A combination of MSCAA (Medicine), Practique™ (Physician Associate) and Examsoft™ (Dentistry) platforms were used across the School of Medicine, Medical Sciences and Nutrition. As each platform has a different set of operating parameters, examinations had to be modified in each case to suit the platform. For example, using the MSCAA platform, only a mark of 0 or 1 can be awarded, while a mark out of 2 would usually be given, with a half‐mark of 1 given as an option. Being a condensed 2‐year course, the Physician Associate programme was most severely affected. The national final examination was cancelled and students offered NHS bank hours until examinations resume. Cadaveric images from Anatomy TV ((™) Primal Pictures) were used under licence. This had been put in place in advance of the lockdown.

Existing, self‐directed, supported workbooks (Findlater *et al*., 2012), which support all anatomy practical classes, were converted into e‐workbooks. These have cadaveric online content, form Acland's Video Atlas of Anatomy and again, Anatomy‐TV linked, where there would previously have been a cadaver or a labelled specimen available for examination. Radiological images were relatively easy to put online, and online versions of current face‐to‐face teaching with expert clinical input were developed. These were augmented with linked existing bespoke 3D photogrammetry material, which can be delivered through the Medical School's bespoke VLE (MyMBChB). Online Panapto™ lectures were delivered at the usual times. In addition, Blackboard Collaborate^TM^ was used to host small group, interactive sessions during timetabled practical class slots, to enable face‐to‐face discussion of areas of difficulty and misunderstanding, and to provide elements of essential discourse. Regular, formative quizzes were offered through the Moodle^TM^ platform. All content was linked from the bespoke MyMBchB platform. The musculoskeletal system, which was taught after the Easter vacation, was an important test bed for online anatomy teaching. In the longer term, it will be desirable for students to catch‐up on the experience of handling and dissecting cadaveric material, but the timing and practicalities remain to be determined. For surgical skills courses, blended courses continued to be held online, with completion of the practical, workshop components deferred to whenever possible in the future. Equality of internet access, presumed to be a key issue for students, has also been a significant issue for many staff residing in rural Aberdeenshire.

### 4.2 Cambridge

For the first year medical course, hands‐on cadaveric dissection was completed in the first two terms of the academic year, with the third term being reserved mainly for revision sessions. These would previously have comprised prosections but now consist of online images and videos, some of which had already been made during class demonstrations throughout the year. As the cadaveric dissection course had been complemented by the Visible Body® 3D Human Anatomy Atlas for all medical students, as well as tutorials created using the VH Dissector Touch (Touch of Life Technologies, Inc.) software throughout the year, the transition to online course provision was not too difficult for students. In addition, enrichment sessions with talks on the history of anatomical dissection, the process of body donation and embalming methods were held using the screen sharing function on the Zoom platform (Zoom Video Communications, Inc.). These sessions also used Breakout Rooms to divide students according to donor table groups for discussions of causes of death and end of life trajectories, facilitated by Year 5 clinical students and anatomy demonstrators. Anatomy revision sessions based on specific clinical scenarios for Year 4 and 5 clinical students were delivered online. Second year anatomy examinations had already taken place prior to lockdown, and the precise online mode of first year summative assessments was still being discussed. The University made the Panopto™ system available for lecturers to create video recordings on desktop or laptop devices and deliver them to students via our VLE Moodle platform that students already use. The Cambridge Centre for Teaching and Learning produced a specific guide to moving to online teaching, as well as regular webinars for teaching staff. The move to online teaching provision was definitely a steep learning curve for everyone. Concerns had also been raised regarding the difficulties some students faced in accessing online educational resources due to factors such as domestic circumstances and internet availability, as well as the possibility of compromised mental health due to prolonged isolation.

### 4.3 Dundee

All anatomy teaching moved online immediately after suspension of face‐to‐face teaching. While anatomy provision is heavily dissection‐based, only relatively few dissections remained uncompleted, as closure came shortly before the end of the semester, when revision sessions are often scheduled. Although several practical classes had to be cancelled, some were histology classes that were easily delivered via the existing ‘virtual microscope’ system, normally accessible to students online. A variety of communication tools were swiftly brought into play, including audio PowerPoint presentations to replace lectures, and the use of VLE (Blackboard) to hold classes in Collaborate. All modules were set up in Microsoft Teams, which allows live events with presentations and/or Q&A, meetings for specific groups or the whole class, and group or personal ‘chat’ channels. Students also had access to a wealth of in‐house online 3D anatomy learning models and resources, created by in‐house medical art and other students. The whole university worked on an alternative assessment plan at module level to cater for the fact that written and practical examinations were no longer possible; this plan was completed, approved and communicated to all students on 26 March. In anatomy, several staff took a ‘conveyor belt’ approach to photographing all existing prosections on the final day at work to ensure a good variety of digital images for use in online spotters, which replaced the end of year practical tests. Most written examinations were conducted online, with some in modified formats. For medical students, the School of Medicine made the decision to cancel all examinations and replace them with ‘enhanced formative assessments’ to be held in the next academic year. Final year medical students were permitted to graduate early in order to join the NHS workforce.

### 4.4 Edinburgh

All anatomy teaching at the University of Edinburgh moved online immediately following closure of the campus. The University made available significant resources and support for academic staff to obtain remote access training using both commercial and bespoke/in‐house platforms, including the secure, password‐protected VLE (LEARN), Microsoft Teams and Kaltura Capture. It was decided to take different approaches for postgraduate and undergraduate students. For the former, given the smaller cohort size, lectures were live‐streamed at a mutually convenient time using Skype, with screen sharing by the academic to show their PowerPoint presentation, with slides made available to students in advance. This allowed for real‐time discussion with students, which was felt to be beneficial. As this approach would not be feasible for the larger undergraduate cohorts, LEARN was used to upload pre‐recorded lectures and associated materials, including access to online textbooks and videos. To ensure that a human/personal touch was retained within remote delivery, a short ‘to camera’ introductory video for each session was given by an academic. The transition to online teaching was aided greatly by having an established online Anatomical Sciences postgraduate programme (Kelsey *et al*., 2020), from which it was possible to repurpose materials, and to obtain mentoring and support from academics with experience of delivering online anatomy teaching. None of the online materials contains images of Edinburgh donor material, but comprised only images from published resources.

To date, the main issue that was identified was a low level of student engagement with online resources, with the vast majority of lectures and other material having been accessed by only *c*.25% of the student cohort, even after numerous email and LEARN reminders. As all practical spotter examinations were cancelled, anatomy questions were embedded into online MBChB and Biomedical Sciences papers in the form of both multiple choice and short essay questions. A decision was taken not to attempt replication of practical, laboratory‐based examinations online, or online replacement for hands‐on dissection classes for the MSc student cohort. Similarly, attempts were not made to reconfigure anatomy‐based CPD or commercial courses and activities using online approaches. Early, anecdotal feedback from students that were moved to an online environment, excluding those who are already enrolled on online courses, suggested that they were missing the face‐to‐face contact with Anatomy staff and the ability to physically interact with specimens (Gillingwater, 2008) in the Anatomy Teaching Laboratory.

### 4.5 Glasgow

The crisis started at a time when the bulk of cadaveric Gross Anatomy teaching in Glasgow had already been delivered and dissection had been completed for the majority of students. Had the crisis started 4 to 6 weeks earlier, disruption of cadaveric practical teaching would have been much more severe. Once the crisis is over, dissection experience will be offered to the latter group. In general, practical cadaveric anatomy sessions were replaced by online resources, and students given access to lecture material, with additional explanations and links to existing material available in the public domain. For spotter examinations, images of specimens were used. Where practical cadaveric anatomy sessions were replaced by online resources, students were given access to further electronic reading material and resources, with additional explanations, and links to existing educational material already available in the public domain. This was complemented by interactive live sessions delivered online, as a ‘virtual’ replacement for dissection classes, and also interactive discussions in a virtual learning environment platform (Moodle and Canvas).

### 4.6 Imperial College London

The bulk of preclinical anatomy teaching for the current academic year had been completed before closure of the College. Therefore, the impact of the pandemic on undergraduate anatomy teaching will depend largely on when the College reopens. If ‘normal service’ is resumed for the beginning of the academic year 2020–2021, the effect will not be significant. While there will be problems such as the lack of newly prepared prosections and the backlog of donors to be released for funerals, as well as the many ‘housekeeping’ tasks that would have been carried out in Anatomy Facilities over the summer months, these should be manageable. However, if the College does not reopen fully, considerable reorganisation of the curriculum will be required. This will inevitably involve more online learning and less direct staff–student contact. The College invested heavily in hosting external online resources and developing in‐house ones. Subjects that are practical in nature are being delayed, at least in part, until the pandemic is over. This delay is likely to involve ‘catch‐up’ summer schools in 2021. Final MBBS examinations were held online. The situation regarding postgraduate training remained unclear. Royal College membership is required for entry into specialist training, but on 16 March, the Surgical Royal Colleges announced the cancellation of the MRCS Part B examinations, which contain a substantial anatomy component, until further notice. In addition, no training courses were available to prepare candidates for these examinations. Much uncertainty therefore remained concerning how entry into specialist training would be determined in the next round, in the absence of professional examinations.

### 4.7 Münich (LMU)

On 30 March, the issue of a German Ministry of Health directive regarding medical education provided the legal framework for preparations for a virtual summer term at the medical faculty of the LMU. The measures are expected to be in place until the clause of national importance relating to the COVID‐19 crisis that was issued by the German Parliament on 28 March is withdrawn. The summer term at the LMU started on 20 April with online teaching. The university acquired a one‐year campus licence from the commercial provider *Zoom* for all academic staff and this was used for preclinical teaching. Due to reported security problems, Zoom was only used when no personalised data (i.e. patient data) were transmitted during teaching. For sensitive meetings, a different system, *dfnconf*, was utilised. All medical students, especially those who have passed their first major examination (M1 or Physikum), were asked to apply for a part‐time contract of 19 hours a week at the university hospitals, which are expected to see high numbers of COVID‐19 patients. These students were provided with an adapted curriculum adjusted to their clinical duties.

### 4.8 Nui Galway

There were only 3 weeks remaining in the NUI Galway term which would have ended on 4 April. The two Gastrointestinal and Renal system modules, which are taught to 185 medical students, were affected by the crisis. These are normally delivered in the last six weeks of the second semester and are taught as integrated systems‐based modules with lectures from Anatomy, Physiology and Biochemistry as well as clinical disciplines. They would also include laboratory sessions in Anatomy and Physiology. While 60% of the Gastrointestinal System Anatomy practical components had been completed, the Renal System practical classes could not be held. Three BSc modules comprising 40 students were also affected. For these students, half of the laboratory component for the Gastrointestinal System module could not be completed, as well as half of the Head and Neck module. One Gross Anatomy demonstration session was cancelled for the 24 students taking the second year Podiatry module. Commercial Biomedical Device Gross Anatomy teaching was suspended, as were the remaining Gross Anatomy sessions for the 19 students enrolled on the MSc/Postgraduate Diploma in multidisciplinary Radiology. All teaching was delivered online, generally via VLE Blackboard using Blackboard Collaborate Ultra tool and Audio enhanced Lecture material delivered via Audio PowerPoint or MP4 files. Students were also supported via a host of e‐learning materials: Acland's Video Atlas of Anatomy, Visible Body Atlas of Human Anatomy, OpenStax Anatomy and Physiology, Complete Anatomy, Anatomus, BioDigital Human, BioDigital Studio and IMAIOS. All first year examinations for medical students in the second semester were conducted online in MCQ format, with cadaveric images from donors who had provided consent. In addition, Wolters Kluwer/Lippincott Williams & Wilkins made many of their anatomy texts and resources freely available for use during the crisis.

### 4.9 Oxford

Much of the preclinical anatomy teaching for the current academic year had been completed before the lockdown. However, the final academic term was significantly affected. The longer‐term impact of the pandemic on anatomy teaching will depend largely on when the University reopens. There is optimism for a return to the ‘new normal’ by the start of the academic year in October 2020, but if the situation extends beyond this, the level of disruption will be considerable. If the University does not reopen fully by then, considerable reorganisation of the medical curriculum will be required. Significant resources have already been devoted to developing online teaching, which migrated from a ‘Weblearn’ system to a new ‘Canvas’ application. The way in which online teaching might best be developed is currently being investigated within a broad framework offering a variety of learning resources, including Instant Anatomy and Acland's Anatomy as well as an extensive set of in‐house notes, diagrams and videos to cover the curriculum. Intensive pre‐professional examination preparation courses held in the evening for regional surgical trainees were suspended.

### 4.10 RCSI

Lectures with slides and commentaries were recorded on PowerPoint Show and additionally as MP4 movies, so that the laser dot was visible on Mac computers. A Research Lecturer, an engineer, was the Department ‘Superuser’ and liaised with staff and Information Technology. Bespoke online guides were produced for dissection and histology ‘in‐house’. A bespoke surface anatomy guide was made available as freeware on YouTube (https://bit.ly/RCSISurfaceAnatomy) to all anatomy students (Morris *et al*., 2016). Staff communicated with students via email and conducted online Q&A sessions. Some students preferred recorded lectures, as they could pause and rewind at will. A number of students commented that they missed the personal tuition and 3D aspects of Anatomy Room teaching. Anatomy examinations, first‐sitting and supplemental, took place online in MCQ format, with only pass/fail grades being awarded. Examination dates were postponed and an exceptional third sitting was offered to mitigate against the disruption to students in view of home circumstances and adaptation to distance learning.

## 5 LOOKING TO THE FUTURE

Given the widespread disruption to Anatomy teaching and practice detailed above, the final section of this review discusses potentially important issues that are likely to need addressing as the anatomical community emerges from the COVID‐19 pandemic.

It is perhaps fortuitous that the Anatomical Society has recently completed the process of design, revision and publication of core anatomy syllabi for a range of student populations (e.g. Smith *et al*., 2016; Connolly *et al*., 2018; Finn *et al*., 2018; Holland *et al*., 2019; Matthan *et al*., 2020). These syllabi provide a useful standardised framework for anatomy educators to design and assess the content of courses, whether delivered face‐to‐face or online, albeit with efforts to validate their potential application and usefulness currently ongoing (Smith *et al*., 2019). What remains unclear is the extent to which online replacement of anatomy teaching, or the uptake of blended learning models (combining on campus with online approaches), will leave unavoidable gaps in core content, knowledge and practical application. This issue will need to be investigated in significant detail over the coming months and years, together with the longer‐term impact on student knowledge and professional capabilities. Whilst the move to online teaching is going to affect all subjects to a greater or lesser degree, the loss of hands‐on practical teaching using cadaveric material is of particular importance and relevance for the study of Anatomy. Early, largely anecdotal, experience suggests that the online resources and opportunities being made available at short notice are not capable of replacing the face‐to‐face, practical‐based experience of an anatomy teaching laboratory. It will, therefore, be important to address how students that have been affected by the COVID‐19 pandemic can replace or substitute these activities in the future, taking into consideration their own concerns and views. Such factors may also influence the decision‐making process for students considering applying for enrolment on courses with an anatomical component over the coming years.

As a group, the authors hold the view that hands‐on examination of cadaveric specimens, and where possible dissection, remains the gold standard for anatomical education. Such activities, with associated benefits concerning student engagement, cannot be replaced or substituted for by virtual/online methods alone. Moreover, considerations regarding the value of working with cadaveric specimens in terms of developing necessary professionalism and manual dexterity need to be considered, when students are missing the opportunity to have a platform for developing and practising empathy, hand and teamwork skills, as well as an appropriate professional attitude. The cancellation of practical‐based examinations is also a concern. When such methods of assessment have been largely removed, with no detriment to student progression in many cases, it is important to try to assess students' practical skills and knowledge using other robust methodologies. In the long term, therefore, reinstatement of practical‐based anatomy examinations will be one of the most important elements of resuming ‘normality’ once the pandemic is over. This situation is likely to be particularly pertinent with regards to surgical trainees, since Royal College Membership is required for entry into specialist training, but at present, the Royal Colleges have suspended their MRCS Part B examinations (which by definition contain a substantial anatomy component) until further notice. This raises significant concerns in terms of the progression of trainees to surgical training with inherent workforce planning implications for the delivery of front‐line medical care. Taken together, it is clear that the short‐ and medium‐term consequences of COVID‐19 disruption for the assessment of anatomical knowledge and skills will need to be addressed by targeted, quantitative research studies over the coming months and years.

Given the importance of cadaveric donor material for all levels of anatomy teaching (undergraduate, postgraduate and CPD), the long‐term supply of donors is of concern. There will need to be national/international guidance on the requirements to add coronavirus to other existing risk factors (e.g. prion/BSE, HIV and TB) as potential post‐mortem risks for both staff and students. Given that a number of institutions rely on imported anatomical material to meet demand, the development of an internationally recognised framework and reporting procedure (as well as best practice guidelines) will be important. Moreover, the resumption of bequeathal programmes will be important in order to meet demand for anatomy training over the coming years. Many universities and medical schools continue to receive several enquiries a day from donors and/or their relatives, despite the closure of body donation programmes. Fortunately, most potential donors do appear to understand that the cessation is temporary. Nevertheless, there is likely to be a decrease in donor acceptance rates due to COVID‐19 as a cause of death and the increased mortality during this period, which may result in a decreased death rate among the donor base in future years. Therefore, when the pandemic is over, raising public awareness of the continuing need for donors for Anatomical Examination may be necessary. It should be noted that some elderly potential donors have been distressed at the thought they will not be able to complete their lifelong intention to donate their body for Anatomical Examination. Anatomists, as a community, must not forget what an important decision this is, both for them and their families.

Several options exist to deal with the issue of donor availability. It is possible, where facilities and skills are present, to introduce or reintroduce longer‐term preservation techniques, such as plastination. This serves to increase the ‘shelf‐life’ of specimens and may also permit future sharing of resources between anatomy facilities, as long as traceability and secure transport facilities can be ensured. Such activities may be supplemented and supported by the use of emerging 3D printing technologies to generate anatomical ‘specimens’ from tomographic radiological data from donor or patient material. It may also be necessary to prioritise embalming of donors that would previously have been used for fresh frozen work. Although the response of the SARS‐CoV‐2 virus to embalming and fixation is currently unknown, the wide range of embalming techniques available offers a good chance of finding at least one that can render COVID‐19 donor material safe for anatomical examination. Of course, the process of embalming will still remain a high‐risk activity for anatomy staff. Information and guidance papers are being published rapidly as the international community gains more experience and knowledge of the virus (e.g. Finegan *et al*., 2020; Kampf *et al*., 2020; Royal College of Pathologists, 2020), and the anatomy community will also be informed by these. Regardless, COVID‐19 testing facilities may be required for anatomy mortuaries, as well as the provision of full PPE for all staff undertaking embalming activities.

## 6 CONCLUSIONS

The United Nations has described COVID‐19 as the most significant event since the Second World War. Things will never be the same again. However, it affords both challenges and opportunities. One opportunity moving forward is for the anatomical community to cooperate more effectively and share resources (both physical and intellectual) more widely. Cooperation will be required to define best practice guidelines for embalming to deal with this new infectious agent. We also need to develop a protocol for dealing with future pandemics that will enable us to respond faster and better than at present. The current situation similarly presents an opportunity to test rigorously the strengths and weaknesses of online anatomical teaching in practice.

Anatomy has been at the heart of medical and scientific teaching and research for several centuries. There is no reason for this not to continue into the future. The strength and willingness of anatomists (including support staff as well as academics) to rise to the challenges that have presented has been a source of great pride within and outside the anatomical community. This is something that we all need to celebrate and recognise.

## CONFLICT OF INTERESTS

The authors have no conflicts of interest to declare.
